# Mycofilters and the Effectiveness of Mycofiltration in the Removal of Contaminants in Water—A Systematic Review

**DOI:** 10.3390/jof12050376

**Published:** 2026-05-18

**Authors:** Sanele Michelle Mnkandla, Patricks Voua Otomo

**Affiliations:** 1 Ecotoxicology Research Group, Department of Zoology and Entomology, Faculty of Natural and Agricultural Sciences, University of the Free State, Qwaqwa Campus, Private Bag x13, Phuthaditjhaba 9866, South Africa; OtomoPV@ufs.ac.za; 2 Ecotoxicology Research Group, Department of Applied Biology and Biochemistry, National University of Science and Technology, Bulawayo P.O. Box AC939, Zimbabwe; 3 Centre for Global Change, University of the Free State, Qwaqwa, Private Bag x13, Phuthaditjhaba 9866, South Africa; 4 South African Environmental Observation Network, 1 Peter Brown Avenue, 16, Pietermaritzburg 3201, South Africa

**Keywords:** fungal remediation, mycelium, mycoremediation, biofiltration, review

## Abstract

Mycofiltration uses saprophytic fungi immobilised on dead organic matter to treat contaminated water. This systematic review aimed to collate literature on mycofiltration, identify water sources subjected to mycofiltration, types of fungi employed, contaminants removed, and removal efficiencies (R%). Articles written in English between 1990 and 2023 were collected from various sources, screened based on inclusion criteria, and critically appraised. Metadata were extracted, and a narrative synthesis was conducted. Forty articles representing 156 studies passed appraisal, with 116 from journal articles, 24 from theses, and 16 from reports. Synthetic stormwater and real wastewater were the most frequently mycofiltered. Fungi of the *Pleurotus* genus were predominantly used in creating mycofilters. Organic contaminants removed included pharmaceuticals and pesticides, with R% between 60% and 100%. *E. coli* was the most studied microbial contaminant, and R% of 30%, 60%, and 90% were reported. Inorganic contaminants were mostly metals with R% above 60%. Overall, contaminant removal by mycofiltration varied, but the technology remained a promising tool. Research gaps observed included a lack of standardised methods for mycofilter preparation and design and little to no assessment of mycofilter saturation. Addressing gaps could aid in increasing mycofilter efficiency and reliable upscaling of mycofiltration.

## 1. Introduction

Good water quality influences the health status of an ecosystem [1,2]. The presence of contaminants in water sources (e.g., ground and surface waters) results in the deterioration of water quality; thus, remedial interventions are of paramount importance. Contaminants such as metals, pesticides, bacteria, pharmaceuticals, and emerging contaminants typically emanate from waste generated from anthropogenic activities such as mining and agriculture, as well as poorly treated wastewater released from hospitals, industries, and wastewater treatment plants (WWTPs) [3,4,5]. Studies have shown that microbial biomass in both natural and engineered systems plays an integral role in the remediation/removal of toxicants [6]. Fungal bioremediation, i.e., mycoremediation, has been reported to be an effective biotechnological tool for eliminating a range of contaminants from the environment [7].

Fungi are eukaryotic organisms that can exist as single cells (such as yeasts) or as long chains of cells (filamentous fungi) [6]. Fungi have multiple ways of eliminating toxicants/pollutants, and these include the secretion of a wide range of extracellular enzymes like laccases and peroxidases, which degrade and transform pollutants, as well as biosorption, a passive process where contaminants are bound following interactions with the fungal mycelium surface. Bioaccumulation is an energy-dependent process involving the active uptake of pollutants into the cells for storage and/or intracellular biotransformation [8,9,10].

Mycoremediation of contaminated water can be conducted by means of fungal enzymes, as crude extracts or purified enzymes, free mycelia in suspension, mycelial pellets, and fungi immobilised on a support such as silica–alginate and loofah sponges [11,12]. The immobilisation of fungi, specifically on dead organic matter, has been used as a technology known as mycofiltration. The fungi employed are saprophytic in nature; thus, the support material also serves as a food source. The mycofilter used in this technology is typically comprised of a unit layered with dead organic matter (i.e., the substrate) and saprophytic mycelia [13]. The mycelia then grow throughout the unit, forming a network of filaments that can treat contaminated water [14].

Creating mycofilters follows a simple process whereby the substrate is first pretreated, e.g., through pasteurisation, prior to inoculation with the saprophytic mycelia [13]. Mycofilters are typically packed/loaded in bowls and buckets perforated underneath, burlap sacks, columns, and jars [1,14,15,16,17]. Contaminated water can be treated following a continuous flow process, i.e., flowing from the top of the mycofilter to the bottom, or via a batch process, where the mycofilter is in contact with the water for a set period, after which the mycofiltered water is removed and a new water sample is introduced [15,17].

Mycofiltration has proven effective in eliminating microbial pathogens from stormwater, removing pharmaceuticals in wastewater, and cleaning drinking water contaminated with heavy metals [1,14,18,19]. Mycofiltration technology thus has great potential as an intervention strategy in removing organic, inorganic, and microbial contaminants.

The objectives of this systematic review were to collate existing research on mycofiltration and ascertain its efficiency in removing specific contaminants from water, wastewater effluent, and other aqueous solutions. The review also ascertained any knowledge gaps for future primary research areas, highlighting the range of mycofiltration applications to date, and potentially aiding in advancing and fostering bioremediation interventions.

The review questions and components of the question are outlined below.

Main question: What is the effectiveness of mycofiltration for removing contaminants from water and other aqueous solutions?
Secondary question: What contaminants are removed by mycofiltration?
Components of the question:
Population: Any water bodies and/or aqueous solutions contaminated with organic, inorganic, and microbial pollutants.
Intervention: The use of saprophytic mycelia in the mycofiltration technology to filter out contaminants in water.
Comparator: Control with no intervention (i.e., no biological filter).
Outcome: Types and levels of contaminants removed in mycofiltered water.

## 2. Materials and Methods

### 2.1. Deviations from the Protocol

Due to the heterogeneity of the studies, a meta-analysis could not be performed, and thus a narrative synthesis of the systematic review was conducted. The systematic review was conducted following the Collaboration for Environmental Evidence (CEE) guidelines [20] and the methods detailed in the systematic review protocol [21]. The review also conformed to the PRISMA 2020 guidelines while incorporating principles from the ROSES checklist to address the environmental nature of the evidence on mycofiltration.

Regarding the search for articles, the search period was adjusted to cover between 1990 and 2023, as the systematic review was conducted in 2024. The supplementary search (citation tracking) was performed only on the full-text articles that met the eligibility criteria to ensure a high return of relevant articles. The comprehensiveness of the search was evaluated against its ability to return 8 benchmark articles that adequately met all the eligibility criteria. Regarding the eligibility criteria, eligible intervention was restricted to saprophytic mycelia immobilised on organic matter, as this is the standard definition of a mycofilter. For study validity, two parameters were removed, namely the risk of confounding variables and the risk of misclassification bias. In the former parameter, the variables stated as confounding were in fact effect modifiers. In the latter parameter, misclassification bias is relevant for observational studies, and this review comprised only experimental studies. A study validity assessment exercise was conducted; however, due to the low evidence base, no studies were excluded based on validity. Regarding data coding for data extraction, information on the mass of the mycofilter used in a study was extracted.

### 2.2. Article Search

The search strategy was followed as described in the protocol, and the detailed search strings are listed in Supplementary File S1. The search was conducted in English with no restrictions in geographic area between the years 1990 and 2023. The search was conducted in the ScienceDirect and Scopus databases on 24–30 May 2024, while searches in Web of Science and Google Scholar were done on 2–6 June 2024. Access to these databases was facilitated by the University of the Free State library (electronic resources). The search string used in the Science Direct database was modified (Supplementary File S1), as the wildcards previously reported in the protocol [21]) were not supported. This modification involved replacing each wildcard with full words (e.g., myceli* = mycelia, mycelial, mycelium). As a result, a total of 15 search strings were employed (Supplementary File S1). A search in Open Access Theses and Dissertations and the websites from the organisations listed in the protocol was carried out on 10–13 June 2024. Searches on specialist websites, as well as the social and research networking sources listed in the protocol [21]), were conducted on 11–13 June 2024. All keywords and hashtags used on specialist websites, social media, and research networking sources are listed in Supplementary File S1. All articles were exported to EndNote X7 reference manager, and duplicates were removed manually.

The supplementary search (i.e., backward and forward citation tracing) was only conducted on full-text articles that met the inclusion/eligibility criteria described in the following section. As outlined in the protocol, the comprehensiveness of the search was evaluated against its ability to return 7 key scientific papers of known relevance to mycofiltration (see Supplementary File S2).

### 2.3. Article Screening and Study Eligibility Criteria

#### 2.3.1. Screening Process

Article screening was done in two sequential stages: title and abstract, then full-text screening. To ensure consistency and clarity in the title and abstract screening, a random subset of 197 articles (10% of the total records) was checked independently by the reviewers SM and PO. Disagreements or ambiguity were resolved through dialogue, and a kappa score above 0.6 indicated agreement between the reviewers.

After the first stage of screening, full texts of the included articles were screened, and only primary data that met the eligible population, intervention, comparator, outcome, and study type were considered. Reviewers did not screen articles they authored, but enlisted a scholar, NK, with expertise in the subject to adjudicate. Consistency between the two reviewers (SM and PO) was assessed independently by screening a random set of 72 articles (20% of the full-text records), and any differences were resolved through discussion. Agreement between reviewers was denoted by a kappa > 0.6.

Articles for which we did not have access to the full text (n = 46) were excluded from this review. For any theses and dissertations from which publications were produced, only the publications were included.

#### 2.3.2. Eligibility Criteria

Studies written in English and published between 1990 and 2023 were eligible for inclusion in the review. The studies also had to meet the following inclusion criteria.

Eligible population: Eligible studies had to focus on contaminated/polluted water and/or effluent and/or aqueous solution/media.

Eligible intervention: Eligible studies had to assess/investigate mycofiltration in removing contaminants/pollutants in water and/or effluents and/or aqueous solution/media. The choice of the fungal mycelia employed was restricted to saprophytic species. As our review focuses on the use of mycelia immobilised on a substrate (lignocellulolytic, agricultural waste, etc.), only such studies were considered.

Eligible comparator: Eligible studies had to show comparison to the control without the intervention (i.e., no mycofiltration).

Eligible outcome: Eligible studies had to report on the types and concentrations of contaminants/pollutants removed in the mycofiltered water/media.

Eligible study types: Both field and laboratory studies that implemented mycofiltration and showed comparison to the comparator.

#### 2.3.3. Study Validity Assessment

Critical appraisal on the studies deemed eligible was conducted, and studies were assigned a low, medium, or high risk of bias. CEE Critical Appraisal Tool version 0.2 (prototype) [22] modified to our review question was used to assess internal validity. The criteria used to evaluate each study are presented in Table 1.

Each parameter was assigned a ‘low’, medium’, or ‘high’ validity based on the outputs listed in Table 1. The overall judgement on the validity of a study was determined as follows: (a) high validity on all parameters was considered of high validity, (b) medium validity in at least one parameter, but not low validity for any other parameters was considered of medium validity, (c) low validity in at least one parameter was considered of low validity. External validity was assessed during full-text screening using the eligibility criteria described in the previous section.

One reviewer conducted the critical appraisal exercise, and the other checked and validated the decisions. Reliability was tested following the test–retest approach. Briefly, after conducting the appraisal, the exercise was repeated after 7 days.

### 2.4. Data Extraction and Coding Strategy

For each screened article that fit the inclusion criteria and study validity, metadata (i.e., descriptive data) and study findings were extracted (Supplementary File S3). The data were extracted according to the codes as shown in Table 2, namely bibliographic information, study location, study site, seasonality, water source, fungal species, substrate employed, type of contaminants removed, mycofiltration process duration, mycofilter unit design and set-up, reported levels of contaminants pre- and post-mycofiltration, and comparator. One reviewer carried out the extraction, and the other cross-checked.

An article could report on more than one experiment; thus, the data were extracted on as many lines. Each experiment was treated as an individual study (e.g., for an article with 3 experiments, the experiments were referred to as study 1, study 2, and study 3). Studies were numbered chronologically as outlined in the data extraction file (Supplementary File S3). For an experiment to qualify as a study, the following factors were considered: water source, fungus species and substrate used, mycofiltration duration and flow rate, mycofilter design, and set-up. For studies in which the mycofilter unit design and/or set-up was sequential batch or continuous with samples collected at different time points, the removal efficiencies were summed and the average calculated and recorded in Supplementary File S3. If a study reported contaminant removal efficiency, efforts were not made to determine the pre- and post-mycofiltration levels/concentrations (unless reported).

### 2.5. Review Synthesis

The PRISMA 2020 flow diagram provided an overview of the reviewing process. After data extraction from all eligible studies, a narrative synthesis was conducted. Information was summarised and visualised in figures. Summaries were descriptive, outlining the distribution of mycofiltration studies, population, fungal species employed in the intervention, mycofiltration process duration, and mycofilter set-up. To observe the effectiveness of mycofiltration in contaminant removal, heat maps were generated using GraphPad Prism 8.0.2, outlining the contaminants removed in each study and the corresponding removal efficiencies (R).

### 2.6. Potential Effect Modifiers

The following potential effect modifiers were identified from the available data:

*Mycofiltration procedure duration.

*Mycofilter pretreatment.

*Substrate used in mycofilter production.

*Fungal species.

*Mycofilter set-up.

*Mycofilter design.

*Size/mass of mycofilter.

*Water source subjected to mycofiltration.

*Water sample pH.

*Temperature.

These possible effect modifiers were identified by both review team members and were considered the main reasons for heterogeneity in the review, covering key aspects relating to producing mycofilters and the various elements involved in the mycofiltration process.

## 3. Results

### 3.1. Literature Search and Screening

**Figure 1:**
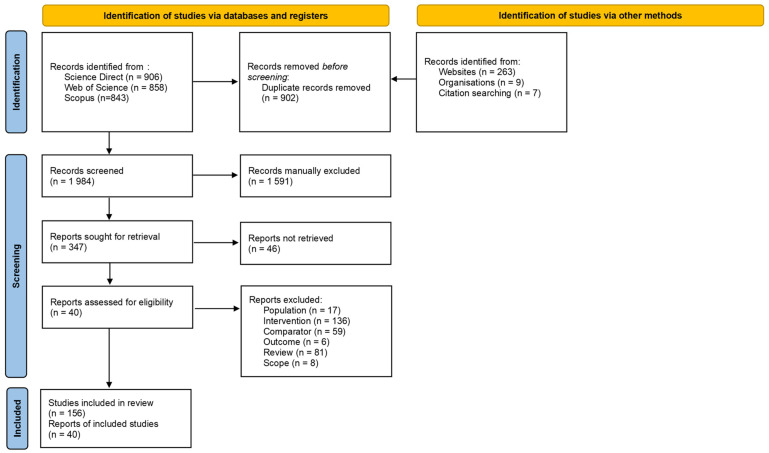
PRISMA 2020 flow diagram. An outline of the process of searching, screening, and synthesis for the systematic review.

The search resulted in the collection of a total of 2886 articles (Figure 1). The search in ScienceDirect returned numerous duplicates due to the modified search strings used. In the Open Access Theses and Dissertations database, the advanced search using search strings could not be performed, as an error message stating that the server was being overwhelmed would appear, despite numerous attempts. The search could only be done using keywords.

Search results from Google Scholar were sorted according to relevance, and only the first 300 results were screened for eligible literature. From the six organisation websites searched, only three organisations, namely Fungi Perfecti, Hab Research, and The Water Network, returned relevant information. Of the social and research networking sources, only X (formerly known as Twitter) and ResearchGate had relevant literature. When the keywords ‘fungal bioremediation’ were used in ResearchGate, only the first 100 articles were screened for eligible literature. The search in Academia.edu could not be performed, as the site required a subscription to access the content. The search returned all eight of the benchmark articles.

After removing duplicates, title and abstract screening resulted in 393 articles being included, while 1591 were excluded (Figure 1). The kappa coefficient obtained at title and abstract screening was 0.8. During full-text screening, 307 articles were rejected and only 40 accepted (Figure 1), and the kappa score at full-text screening was 0.9. Screening resulted in a high exclusion rate because the search terms were broad, leading to the retrieval of studies irrelevant to this systematic review. The most common reason for exclusion was inappropriate intervention (45%), where remediation was achieved through, e.g., fungal/mycelial suspensions, agar plugs, fungal pellets, and/or fungi immobilised on polyurethane form (PUF) supports. As mentioned in the inclusion criteria, the only eligible studies were those where mycelia were immobilised or cultured on a substrate of ligninolytic and/or agricultural nature. Other major reasons for exclusion were the lack of appropriate comparator (19%) and review articles (27%).

### 3.2. Study Validity Assessment

**Figure 2:**
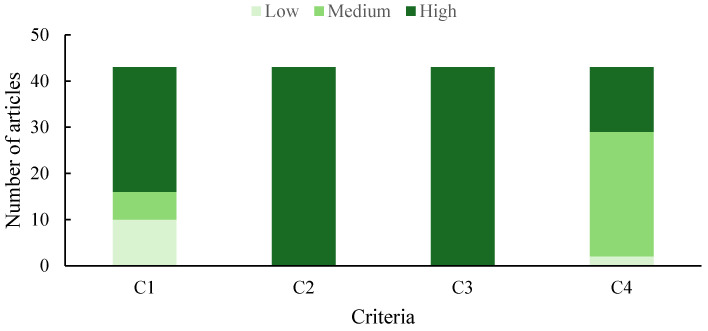
Number of articles with low, medium, and high validity for each study validity criterion (C). C1 = study design, C2 = risk of performance bias, C3 = risk of reporting bias, C4 = risk of analysis bias.

Of the 40 articles that met the eligibility criteria, 11 had an overall high validity rating, while 22 had an overall medium validity rating. Seven articles had an overall low validity rating. Among the assessed criteria, the study design (criterion 1) had the highest number of low validity ratings (Figure 2). Studies within the articles either did not mention the number of replicates or had no replicates in their study design.

### 3.3. Sources and Distribution of Mycofiltration Studies (Descriptive Statistics)

**Figure 3:**
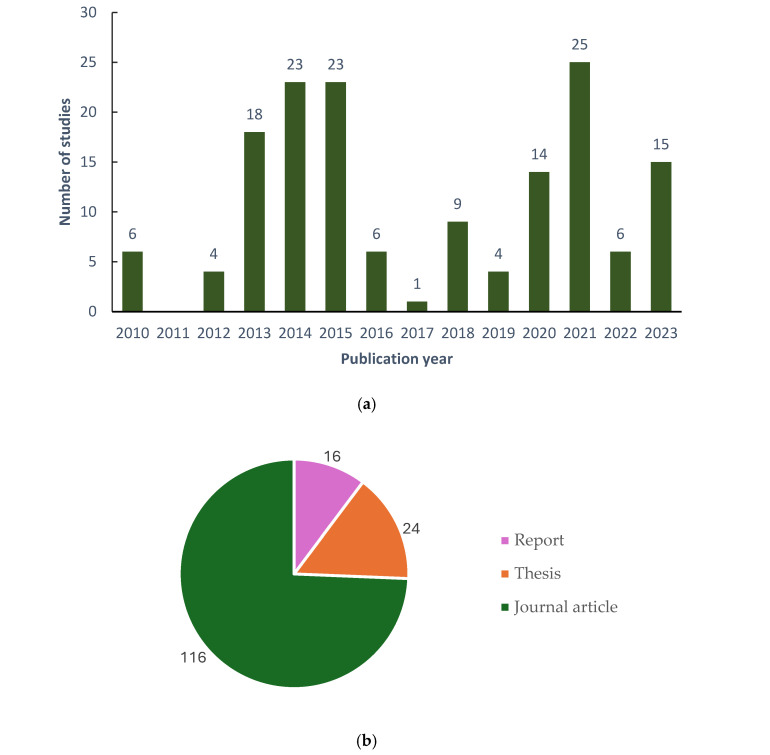

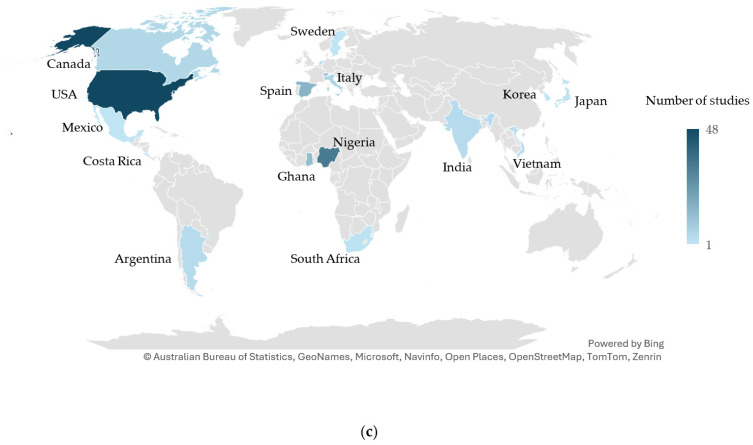
Temporal and geographic distribution of mycofiltration studies. (**a**) Number of studies per year, (**b**) sources of mycofiltration studies, and (**c**) geographic distribution of mycofiltration studies.

The present review aimed to consider articles published between 1990 and 2023; however, only articles published between 2010 and 2023 passed the eligibility screening. From these articles, 156 studies were observed (Figure 1). Studies on mycofiltration first peaked in the years 2013, 2014, and 2015, with 18 studies in 2013 and 23 studies each in the years 2014 and 2015 (Figure 3a). As time progressed, a second peak was observed in 2020, 2021, and 2023 with 14, 25, and 15 studies, respectively (Figure 3a). Of the 156 studies, the majority, i.e., 116, were from published journal articles, whilst 24 were from master’s and doctoral theses and 16 from technical reports (Figure 3b). All studies were conducted in the laboratory, with the authors reporting the findings and the controlled conditions under which the experiments were conducted.

In terms of geographic distribution, mycofiltration studies were conducted across five continents (Figure 3c). In North America, mycofiltration studies were conducted in the USA (48) and Canada (5) (Figure 3c). Single studies were conducted in North America (Mexico) and Central America (Costa Rica), and four studies were reported in South America (Argentina) (Figure 3c). In Africa, mycofiltration studies were performed in Nigeria (n = 33), Ghana (n = 13), and South Africa (n = 2) (Figure 3c). Mycofiltration studies in Europe were conducted in Switzerland (n = 12), Italy (n = 6), Spain (n = 16), the Netherlands (n = 4), and Sweden (n = 1). In Asia, India and Vietnam conducted four and three studies, respectively, whilst one study was reported in South Korea and two in Japan (Figure 3c). Despite continents like Africa, Europe, and Asia being represented, there are still many countries within these regions where mycofiltration has not been investigated (Figure 3c). Strikingly, no mycofiltration studies have been conducted in the Middle East or Australia (Figure 3c). These findings highlight how mycofiltration technology is lagging.

### 3.4. Water Sources Used in Mycofiltration

**Figure 4:**
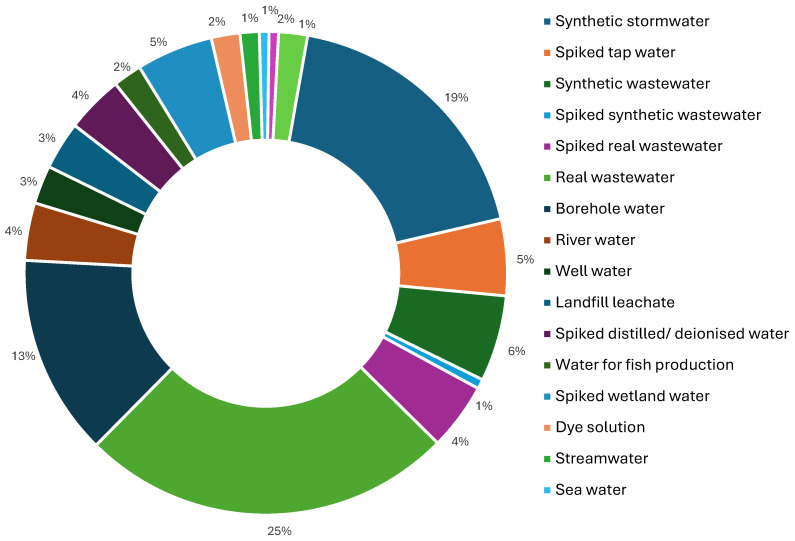
Proportions of studies (%) depicting water sources subjected to mycofiltration.

Regarding the population component of this review, various water sources were employed in the mycofiltration studies (Figure 4). Synthetic stormwater and real wastewater were the most frequently used water sources in mycofiltration studies, comprising 19% and 25% of the total studies, respectively (Figure 4). Synthetic stormwater was typically prepared by dissolving salts such as sodium thiosulphate, calcium chloride, and ammonium chloride in deionised water prior to the addition of the contaminants of interest. Regarding real wastewater, studies used treated and/or untreated wastewater collected from municipal wastewater treatment plants (WWTPs), agricultural drainage channels, slaughterhouses, hospital effluent, aquaculture wastewater, and industrial wastewater.

Synthetic wastewater was used in 6% of the studies (Figure 4), and it characteristically consisted of unsterile tap water dissolved with various micropollutants depending on the experiment. Only 1% of the studies used spiked synthetic wastewater (Figure 4). A wide range of natural water sources were also used in mycofiltration studies, and these included bore-hole water (13%), well water (3%), river water (4%), stream water (1.5%), and pond water (1%) (Figure 4). Landfill leachate was also used in 3% of the studies (Figure 4).

### 3.5. Fungal Species Employed in the Mycofiltration Intervention

**Figure 5:**
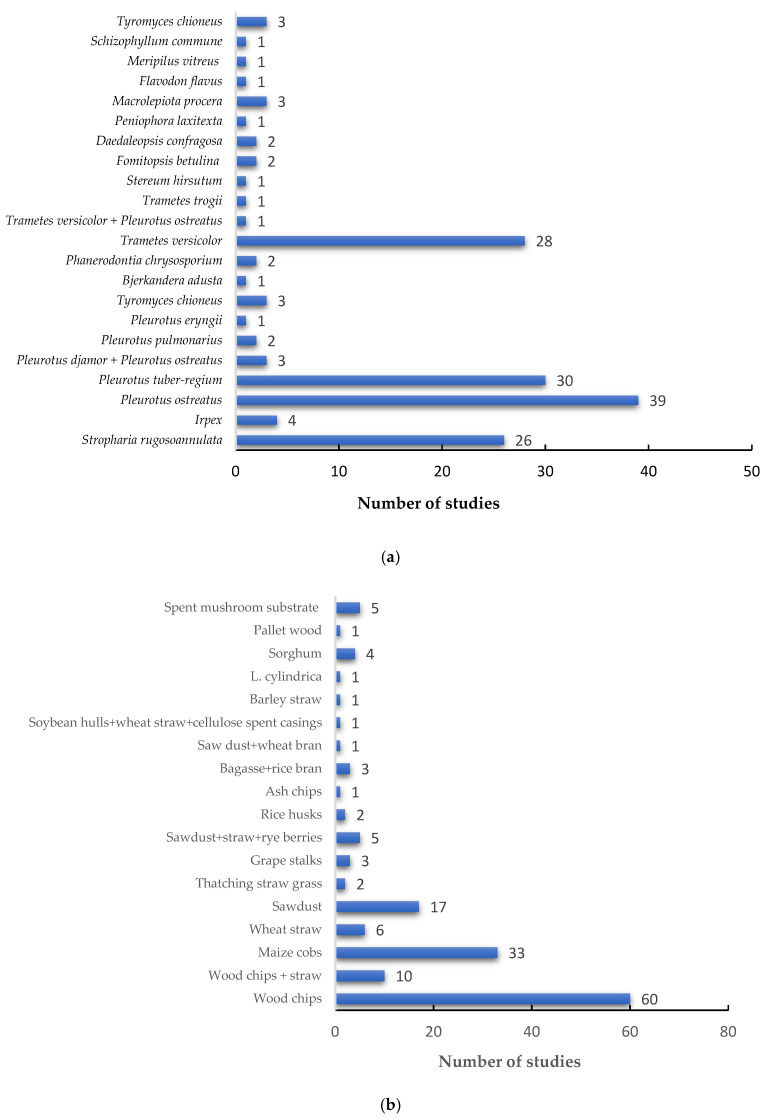
Number of studies: (**a**) fungal species employed and (**b**) types of substrates used for the mycofiltration intervention.

With regard to the intervention aspect of the present review, fungi belonging to the *Pleurotus* genus were predominantly used (Figure 5a). The species *Pleurotus ostreatus* and *Pleurotus tuber-regium* were used in 39 and 30 studies, respectively (Figure 5a). Only three studies used *Pleurotus ostreatus* and *Pleurotus djamor* in combination, whilst two studies used *Pleurotus pulmonarius* and one study used *Pleurotus eryngii* (Figure 5a). *Stropharia* and *Trametes* were the second- and third-most used genera. The species *Stropharia rugosoannulata* was used in 26 studies, while *Trametes versicolor* was used in 28 studies (Figure 5a). Several other fungal genera were used, albeit in fewer studies, and these included *Irpex*, *Tyromyces*, *Bjerkandera*, *Phanerodontia*, *Stereum*, *Fomitopsis*, *Daedaleopsis*, *Flavodon*, *Meripilus* and *Schizophyllum*. To ensure the use of the correct current names in this review, all names were validated using Index Fungorum [23]. Illustrating current name [=name in the original reference], the following were changed: *Trametes versicolor* [=*Coriolus antarcticus*], *Fomitopsis betulina* [=*Piptoporus betulinus*], and *Meripilus vitreus* [=*Physisporinus vitreus*].

Concerning the choice of substrate to cultivate/create the mycofilter, the majority of the studies used woodchips (n = 60), followed by maize cobs (n = 33), sawdust (n = 17), a combination of woodchips and straw (n = 10), and wheat straw (n = 6) (Figure 5b). Other studies used a combination of sawdust, straw and rye berries (n = 5), spent mushroom substrate (n = 5), sorghum (n = 4), bagasse and rice bran (n = 3), grape stalks (n = 3), thatching straw (n = 2), rice husks (n = 2), sawdust and wheat bran (n = 1), ash chips (n = 1), pallet wood (n = 1), and *Luffa cylindrica* sponge (n = 1) (Figure 5b).

### 3.6. Mycofilter Design, Set-Up, and Mycofiltration Duration

**Figure 6:**
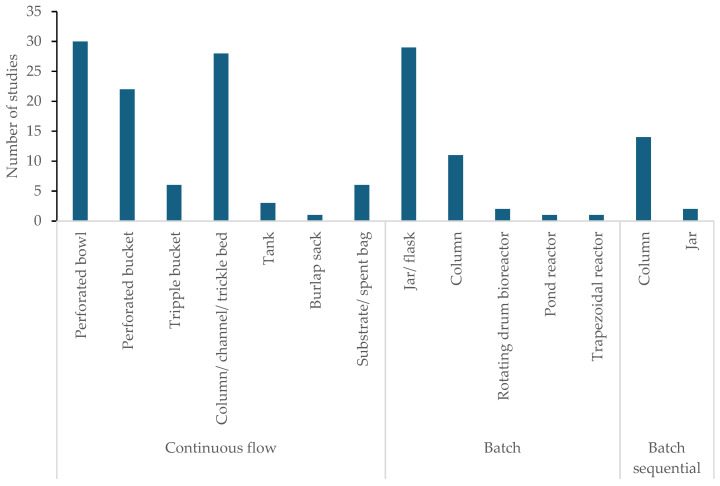
Proportion of studies based on mycofilter design and set-up (continuous flow, batch, or batch sequential).

Mycofilters were designed/packed either in perforated bowls and buckets, loaded in columns, sacks, and flasks/jars, or in some instances placed in specialised bioreactors, e.g., a rotating drum or trapezoidal reactor (Figure 6). Unless specified (e.g., triple bucket, thus three mycofilter units), the studies used a single mycofilter. The sizes of the mycofilter design/system varied depending on the mass of the mycofilter loaded. Where sizes were reported, the buckets were 18.9 L, while columns, which were either glass or plastic, were 12–50 cm in length and 2.5–11 cm in width. The flasks employed were 100 or 500 mL, whilst the jars were 250 mL or 355 mL. The bioreactors were plastic containers of 1.3 L volume or columns of dimensions 50 × 11 cm and 25 × 3 cm. The mass of the mycofilters used ranged between 1 g and 10 kg.

The majority of the studies (n = 96) (Figure 6) used the continuous flow set-up, whereby the water sample is introduced to the top of the mycofilter at specified flow rates (as observed in some instances) and allowed to trickle down. Mycofiltration studies following the batch set-up numbered 44, much fewer than those using continuous flow (Figure 6). In the batch set-up, the mycofilter was typically in constant contact with the same water sample over a period of time. There were 14 studies that followed the batch sequential set-up in column mode, and 2 studies in a jar (Figure 6). The batch sequential set-up meant that the mycofilter was in contact with a water sample for a period, after which the water was removed and the mycofilter left to rest before a new water sample was introduced to the same mycofilter.

**Figure 7:**
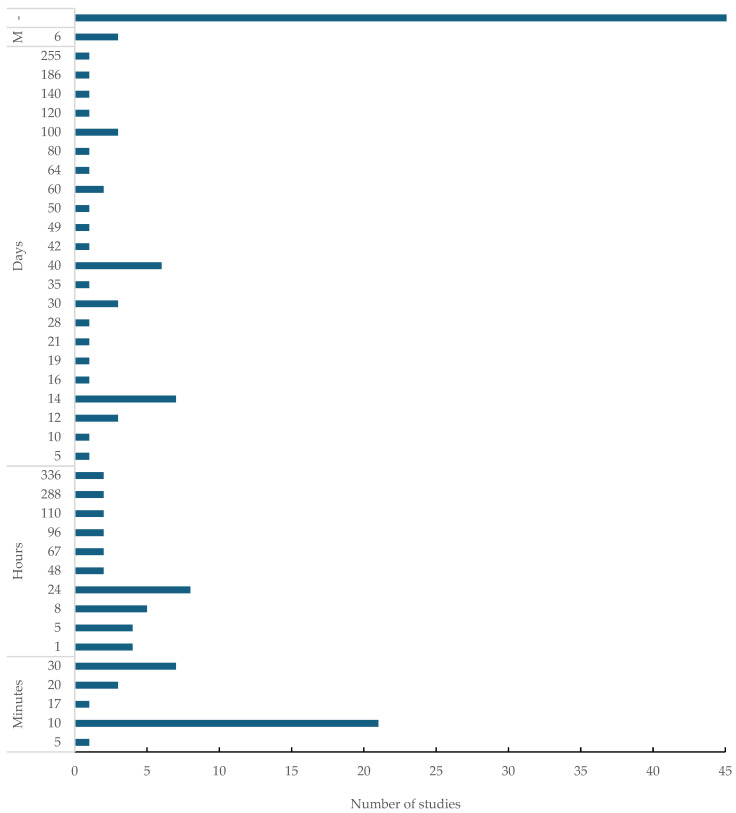
Duration of the mycofiltration process (from start to finish) per study. Total mycofiltration run time in minutes, hours, days, months (M). (-) represents not reported.

Mycofiltration could run for minutes, hours, days, and months (Figure 7). In the minute and hour ranges, most mycofiltration studies were run for 10 min and 24 h (n = 21 and n = 8, respectively) (Figure 7). In terms of days, the majority of the studies (n = 7) conducted filtration over 14 days (Figure 7). Only three mycofiltration studies were run over 6 months (Figure 7). Interestingly, 47 out of 156 studies did not report on the duration of the mycofiltration procedures (Figure 7).

### 3.7. Contaminants Subjected to Removal by Mycofiltration & Removal Efficiencies

**Figure 8:**
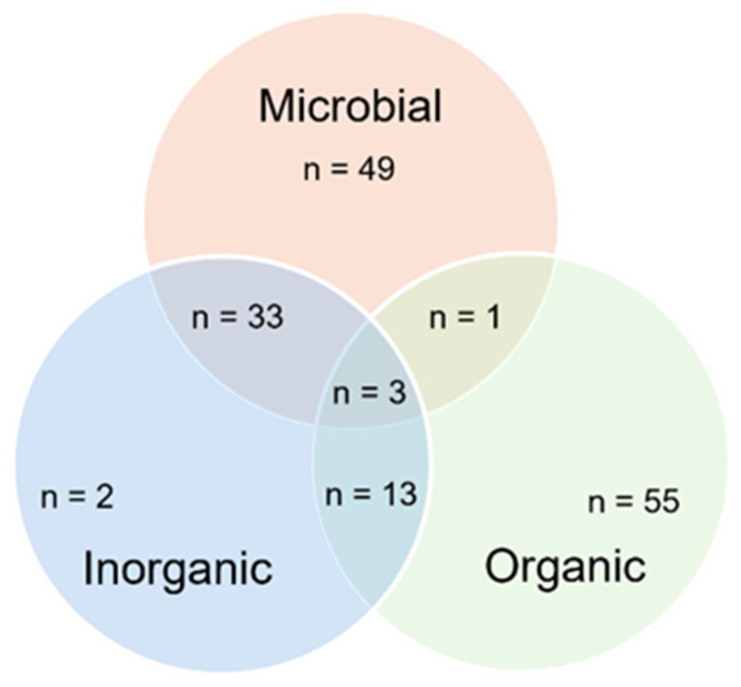
Venn diagram showing the number of studies that focused on mycofiltration of organic, inorganic, and microbial contaminants.

Regarding the outcome component of the present review question, a range of contaminants falling in the microbial, organic, and inorganic classes were removed in the mycofiltration studies considered. Microbial contaminants were studied the most, followed by organic contaminants (Figure 8). Some studies focused on the removal of more than one class of contaminants, e.g., organic and inorganic contaminants, microbial and inorganic contaminants, and microbial and organic contaminants (Figure 8). Only three studies investigated the mycofiltration of all the classes of contaminants (Figure 8).

**Figure 9:**
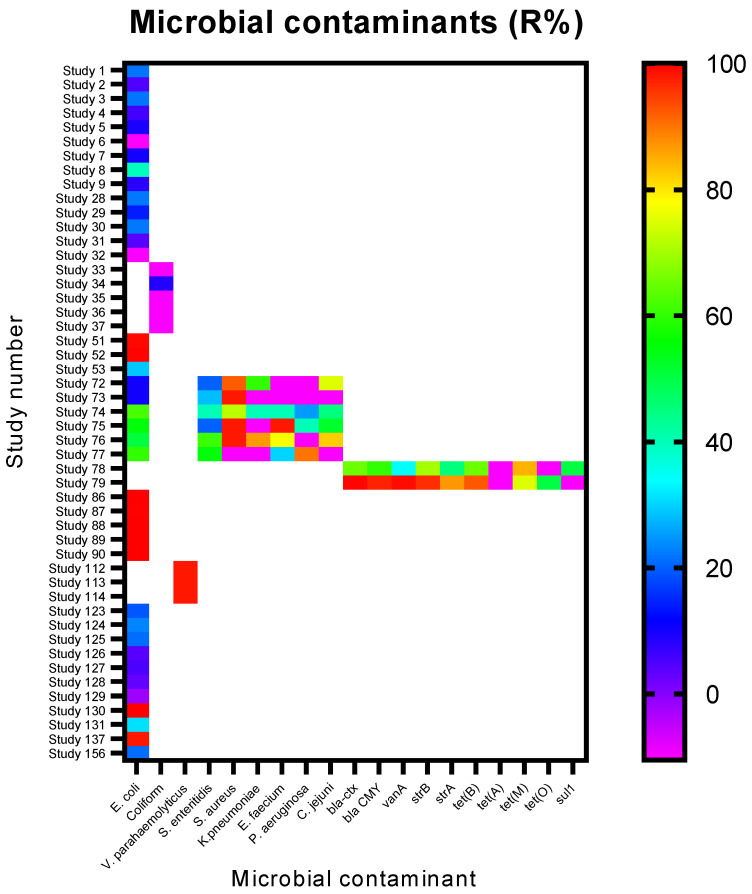
Removal efficiencies (%) of microbial contaminants from the studies included in the present review. White spaces/blocks indicate that a corresponding contaminant was not investigated in a study.

**Figure 10:**
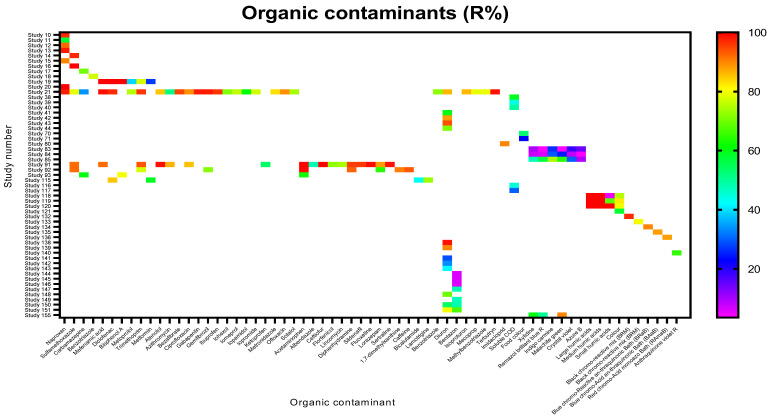
Removal efficiencies (%) of organic contaminants from the studies included in the present review. White spaces/blocks indicate that a corresponding contaminant was not investigated in a study.

**Figure 11:**
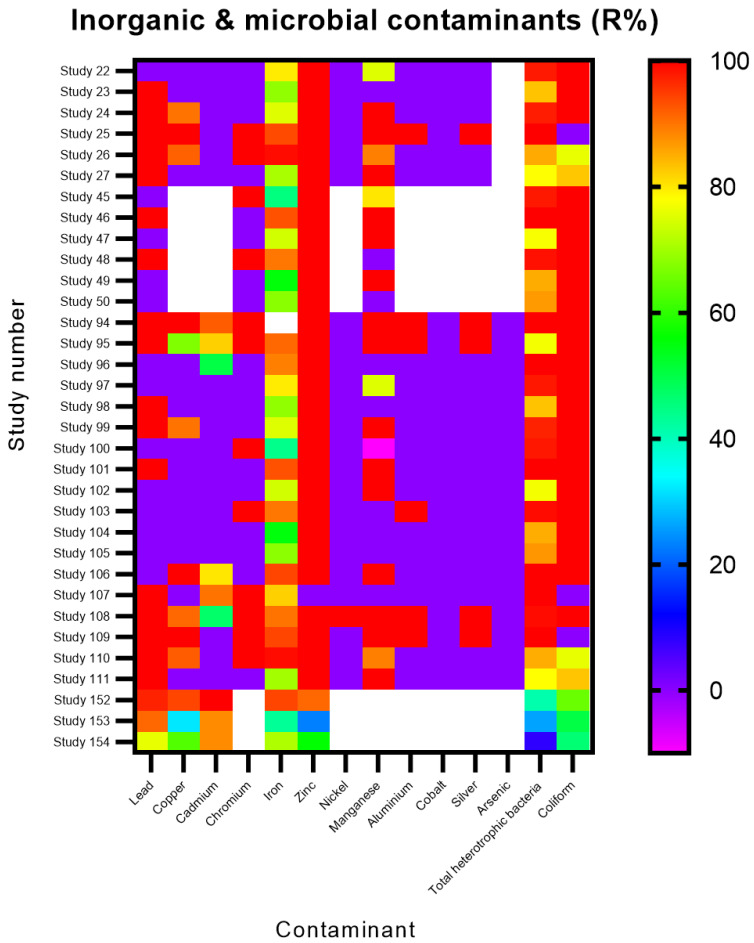
Removal efficiencies (%) of inorganic and microbial contaminants from the studies included in the present review. White spaces/blocks indicate that a corresponding contaminant was not investigated in a study.

The alternative hypothesis tested by the authors was that fungi could efficiently remove contaminants through mycofiltration. The removal efficiencies (R%) for the contaminant classes investigated are presented in Figure 9, Figure 10 and Figure 11. Of the microbial contaminants assessed, *Escherichia. coli* was the most studied (Figure 9). Only 9 out of 39 studies, however, reported a high *E. coli* R% of >90%, while 4 studies recorded 60% removal (Figure 9). The majority of the studies reported much lower *E. coli* R%, i.e., <30% (Figure 9).

In studies focusing on the mycofiltration of a consortium of bacteria, namely *Salmonella enteritidis*, *Staphylococcus aureus*, *Klebsiella pneumoniae*, *Enterococcus faecium*, *Pseudomonas aeruginosa*, and *Campylobacter jejuni*, it was found that *Staphylococcus aureus* was the most efficiently removed bacterium (R% > 70%), followed by *Salmonella enteritidis*, where R% ranged between 20% and 70% (Figure 9). Two studies assessed the mycofiltration of antibiotic resistance genes (bla-ctx, bla CMY, vanA, strB, strA, tet(B), tet(A), tet(M), tet(O) and sul1) present in a wetland within the University of Washington Bothell/Cascadia College Campus, and the most efficiently removed genes were bla-ctx, bla CMY, vanA, strB, strA, and tet(B), with R% ranging between 70% and 100% (Figure 9). Quantitative PCR was used to quantify these genes, which were present in the total DNA extracted from the water samples.

A total of 65 organic contaminants falling under pharmaceuticals, pesticides, dyes, humic acids, and some physicochemical parameters were subjected to mycofiltration across the various studies (Figure 10). In general, all the contaminants, save for a few dyes (indigo carmine, crystal violet, and azure B), were efficiently removed, with R% ranging between 60% and 100% (Figure 10). Interestingly, in studies 83 and 84, removal efficiencies of xylidine, Remazol brilliant blue R, and malachite green were <25%, but were higher in study 155, ranging between 50% and 90% (Figure 10).

Thirty studies investigating the mycofiltration of metals and microbes revealed that all the microbial contaminants (heterotrophic bacteria and coliform) were efficiently removed (R% > 80%) (Figure 11). Regarding the metals, zinc and iron, which were investigated across all studies, were efficiently removed, with zinc demonstrating a higher R% of more than 90%, while iron ranged between 60% and 100%. For the other metals (lead, copper, cadmium, nickel, manganese, aluminium, cobalt, silver, and arsenic), removal efficiencies were generally in the extremes, i.e., R% was either high (>90%) or low (<10%) (Figure 11).

In the studies where inorganic and organic contaminants were investigated, the contaminants of interest were physicochemical, i.e., colour, turbidity, total suspended solids, pH, total dissolved solids, electrical conductivity, dissolved oxygen, biological oxygen demand (BOD), chemical oxygen demand, total nitrogen, and phosphorus [16]. The best R% values were recorded for colour, BOD, and total nitrogen, at 75%, 88%, and 86%, respectively.

In the study that assessed the mycofiltration of both organic and microbial contaminants, i.e., pharmaceuticals, ibuprofen, ketoprofen, naproxen, and heterotrophic bacteria, the removal efficiency was higher than 70%. The studies that focused on the removal of nitrate and phosphate (inorganics) showed that mycofiltration was inefficient, as R% values were negative due to leaching from the substrate. Where inorganic, organic, and microbial contaminants were mycofiltered, there was better removal of the coliform (100%) compared to the metals and physicochemical parameters investigated.

Regarding the comparator, i.e., controls, studies using uninoculated substrate reported contaminant removal, albeit to a lesser degree, compared to the mycofilter. Removal was attributed to the adsorptive nature of the respective organic matter (i.e., substrate). The studies with ‘uninoculated substrate’ and ‘no mycofilter’ controls remained comparable due to the use of similar substrates in mycofilter cultivation. The effect of contaminant removal by uninoculated substrate could thus be inferred in the studies using ‘no mycofilter’ as the comparator.

## 4. Discussion

This review’s findings show that original research articles dominate the publication output regarding mycofiltration. Mycofiltration studies appeared to be dominant during the 2013–2015 period, and interest in the technology generally waned as the years progressed, with a slight peak between 2020 and 2023. Despite North America, Africa, and Europe representing key research hubs in mycofiltration, this technology remains understudied in many other parts of the world, and thus appears to occupy a small niche under the umbrella of fungal remediation. This may be due to the abundance of other fungal bioremediation methods practised by researchers, including the use of fungal suspensions, pellets, and/or fungi immobilised on non-ligninolytic supports [24,25,26,27]. While each of these may have their merits, mycofiltration offers the advantages of low cost and simplicity and thus should be explored more.

Regarding the water sources subjected to mycofiltration, real wastewater and synthetic stormwater were the most studied. Remediation of these water sources is of paramount importance, as they are classified as point and non-point sources of pollution, respectively, that enter water bodies and deposit contaminants [14,28]. Wastewater typically discharged into effluent-receiving water bodies contains a range of organic and inorganic substances, as well as various microorganisms, while stormwater flows over surfaces such as roofs, roads, and agricultural land, collecting contaminants such as pathogens, metals, and pesticides and deposits them in nearby water bodies [29]. The presence of these contaminants has detrimental environmental and health impacts, and thus the need for studies on bioremediation tools such as mycofiltration. While the studying of synthetic stormwater and/or wastewater enabled the authors to work under controlled conditions to optimize contaminant removal, such studies ought to ensure further work is done on real environmental samples to test the applicability of mycofiltration.

As mentioned earlier, mycofilters are a product of solid substrate fermentation, where an organic substrate is colonised by saprophytic mycelia. The findings in this review revealed that fungal species from the *Pleurotus* genus were predominantly used, and this is due to their aggressive growth and high ability to outgrow naturally occurring microorganisms, thus outgrowing competition [18,30]. Various substrates were also used, and regardless of the substrate used, Sen and colleagues [31] stated that it is important that the mycelia are firmly attached to the substrate to ensure that the flowing water during the mycofiltration process does not tear and wash them away. The substrate should also be adequately pretreated through, e.g., pasteurisation, before inoculation with mycelia, to prevent the growth of moulds [31].

Regarding mycofilter design, the present review showed that there are no standard methods employed—from substrate particle size to mycofilter mass (g). This poses a challenge, as this results in variability in contaminant removal efficiency across studies. Particle size, for example, has a significant effect on pollutant removal, as demonstrated by Osarenotor and colleagues [16]. In their study, they observed better removal of wastewater contaminants with particle sizes of 2.36 mm compared to 0.6 and 1.18 mm. While substrates do differ in nature, standardizing particle sizes according to substrate may aid in ensuring efficiency and replicability.

This review revealed the set-ups under which mycofiltration is conducted. The continuous flow set-up employed by most studies has the merit of being low in cost, as it can be performed without energy (i.e., pumps), relying on gravity. It can also be upscaled for broader application [2]. The disadvantages, however, are that due to the heterogeneous nature of the mycofilter, i.e., the layering and position of the substrate and mycelia, channelling (i.e., the preferential path of the water flow) exists. This implies that some parts of the mycofilter remain unused, resulting in underutilisation/reduced capacity of the mycofilter. Another challenge with the continuous flow set-up, where columns are used, is clogging at the bottom, due to mycelia being washed off the substrate as the water trickles down the mycofilter [18,32]. The batch configuration, on the other hand, eliminates the challenges observed with the continuous flow set-up, i.e., preferential water path flow and clogging; however, the set-up requires energy (where agitation is used), and feasibility upon scaling up to, e.g., industrial scale is limited. The authors using the batch sequential configuration cited the advantage of allowing the fungi to ‘rest’, because under complete saturation conditions (i.e., constant contact of fungi with water), the fungi were stressed, and this resulted in short fungal survival [18,32]. Alternating saturated and unsaturated conditions thus ensured longer survival. Despite the alluded advantage, the batch sequential set-up faces a cost challenge, as it requires equipment and energy, such as automated valves to open and close at the bottom of the filter.

Mycofiltration was run over different lengths of time; however, some studies did not report on mycofiltration duration. Not reporting on mycofiltration duration affects the interpretation and applicability of the study findings, as they tend to be inconclusive. The aim of laboratory-based mycofiltration studies is not only to investigate the concept, but also to obtain baseline data that can be used when upscaling. All information thus ought to be available, and mycofiltration duration is valuable data, as it gives an indication of how long the mycofilter can be used and still achieve high removal efficiency before the need for renewal.

All three classes of contaminants, i.e., microbial, organic, and inorganic, were removed by mycofiltration, albeit to varying degrees. Of the microbial contaminants assessed, *E. coli* was the most studied, as it is typically used as an indicator of the bacteriological quality of water [33]. The discrepancies observed in *E. coli* removal efficiency were suggested to be due to factors such as different fungal species and growth substrates having differing abilities to filter *E. coli*, differences in contact time (i.e., between the contaminant and mycofilter), and mycofilter set-up [17,34,35]. In the studies where R% was the highest (i.e., 98–100%), the fungal species and growth substrates used were: (i) *Pleurotus ostreatus* used with either wheat or barley straw or a combination of woodchips and straw [17,36,37]; (ii) *Fomitopsis betulina* or *Daedaleopsis confragosa* or *Pleurotus eryngii* used with a substrate of sawdust mixed with straw and ryeberries [38]; and (iii) *Flavodon flavus* or *Meripilus vitreus* or *Schizophyllum commune* used with a mixture of bagasse and rice bran [39]. A longer contact time and conducting mycofiltration in a batch set-up with agitation improved R%, as observed in studies 51 and 52 and 72–77 (Figure 9) [17,31]. Where R% values were negative, i.e., having an increased amount of contaminant in the mycofiltered media, it was reported to be a result of native non-pathogenic bacteria present in non-sterilised substrate being exported into the mycofiltered media or bacteria present in the background of the water source being filtered [31,34]. Regarding the mechanism of bacterial removal, some studies suggested physical capturing of the bacteria within the hyphae of the mycelium and biodegradation via metabolites secreted by hyphae [31]. Umstead [38] suggested that the hydrogen peroxide secreted by fungi acts as an antimicrobial agent, inhibiting bacteria. Hydrogen peroxide is secreted in large amounts by fungi to depolymerise the growth substrate during colonisation [38]. Regarding antibiotic-resistance genes, the highest removal was achieved using a mycofilter made of *Stropharia rugosoannulata* and woodchips [31].

Organic contaminants were generally removed more efficiently, and a common theme across the studies removing organic contaminants was that high contact time between a contaminant and mycofilter was necessary for efficient contaminant removal. Where the highest R% was reported, the fungal species and substrate used were *Pleurotus ostreatus* with woodchips or *Trametes versicolor* with rice husks or sorghum [5,18,31,32]. The poor removal efficiencies observed with some dyes may be attributed to the choice of substrate used, i.e., grape stalks [40]. As mentioned earlier, the choice of fungal species and substrate influences the efficiency of contaminant removal. In these studies (i.e., studies 83 and 84, Figure 10), low amounts of the biodegrading enzyme laccase were produced, thus translating into reduced remediation activity [40]. Contact time between the mycofilter and the dyes may have also resulted in these discrepancies, as the experiments were run for 5 h in studies 83 and 84 [40] and 24 h in study 155 [41]. In the studies where the intervention was effective on the organic contaminants [42,43,44,45,46,47,48,49,50,51,52,53,54,55,56,57,58], the studies attributed it to adsorption onto the mycelia and/or biodegradation by non-specific enzymes [18,32]. Adsorption is facilitated by functional groups (e.g., hydroxyl, carboxyl and amino groups, etc.) provided by proteins, chitin, and glucan, which make up the mycelial cell wall and bind contaminants using mechanisms such as ion exchange and hydrogen bonding [59]. Most studies also highlighted that physicochemical characteristics, such as pH, affect the adsorption of contaminants into the biomass. The enzymes involved in biodegradation include extracellular enzymes, e.g., laccase, manganese peroxidase, and lignin peroxidase, and intracellular enzymes such as cytochrome P450 monooxygenases and oxidative enzymes [18,32]. In the present review, the removal of organic solvents was not investigated. However, it is important to note that high concentrations of these solvents in contaminated water cause toxicological stress, hampering the action of the intra- and extracellular enzymatic machinery responsible for biodegradation [60]. At high levels of organic solvents, the extracellular enzymes undergo unfolding, i.e., changes in the protein structure resulting in loss of enzyme activity, whilst the intracellular detoxification pathway (transport, metabolism, and catabolism) involving cytochromes is disrupted [61]. Cell growth is also affected, so collectively these effects would negatively affect the efficacy and lifespan of a mycofilter, diminishing the remediation potential of mycofiltration.

Where a mixture of inorganic (i.e., metals) and microbial contaminants was studied, an interesting contrast with the findings from studies that solely focused on the removal of microbial contaminants was observed. It is possible that the fungal species and substrate (*Pleurotus tuber-regium* and maize cobs; *Macrolepoita procera* and corn cobs respectively) used in the studies mycofiltering metals and microbials were optimum for remediation, thus effectively removing the contaminants [1,2,19,62]. It should be noted, however, that these studies did not report the duration of the mycofiltration process. The high R% may thus be due to a measure of the earliest mycofiltered effluents, which would typically be low in contaminant concentrations, as a fresh mycofilter would have more adsorption sites and antimicrobials to trap and degrade contaminants. Fungal remediation of metals is reported to occur via processes such as biosorption onto the mycelial surface as well as through acid–base bioremediation, i.e., where acids secreted by fungi chelate with metal ions and the resulting complex is taken up by the fungi for further detoxification [63,64]. Poor metal R% is generally attributed to a fungal species not being optimal for removing a contaminant and/or the presence of other contaminants with stronger driving forces competing for biosorption onto the mycofilter [10].

This review has several limitations. The search was conducted exclusively in English and may have missed non-English literature that met the inclusion criteria. Despite access to a wide range of research articles, electronic resources, and databases via our institutional library, 46 full-text articles could not be retrieved in this review. The exclusion of all studies published before 1990 may have limited the retrieval of more relevant articles. The inability of Open Access Thesis and Dissertation (one of the identified databases) to accommodate advanced searches using search strings may have led to the omission of relevant theses and dissertations. Having one reviewer conduct the data extraction and critical appraisal exercises and the other only cross-checking was another limitation, which could have introduced bias.

In the present contribution, potential biases within the included studies could have emanated from effect modifiers. The modifiers that influenced removal efficiency could be grouped into three main categories: mycofilter intervention, mycofiltration procedure, and water characteristics.

Regarding mycofilter intervention, the use of different fungal species (e.g., *Pleurotus ostreatus* vs. *Trametes versicolor*) resulted in different contaminant degradation rates or biosorption. Similarly, the substrate used to culture the fungi (e.g., woodchips, maize cobs, wheat straw) most likely impacted the mycofilter’s structure, adsorption properties, and overall performance. Furthermore, the mycofilter design (e.g., perforated bowls, columns, jars, specialised bioreactors) and set-up (continuous flow or a batch configuration) equally influenced the outcome of the mycofiltration process.

The duration of the mycofiltration procedure, which often went unreported, significantly impacted the observed removal efficiency. As for water characteristics, water pH, temperature, and origin (e.g., synthetic stormwater, real wastewater, bore-hole water, river water, etc.) also contributed to variable results from one study to another. These differing experimental parameters, as already mentioned, made it impossible to conduct a meta-analysis because of the heterogeneity in the review, which was to be expected due to the lack of a standardised protocol informing the preparation, design, and application of mycofilters.

Finally, because the 156 studies were all conducted in the laboratory (i.e., under controlled conditions), their geographic distribution in terms of climatic variability could not be ascertained.

## 5. Conclusions

This systematic review aimed to collate research on mycofiltration and evaluate its efficiency in the removal of contaminants. Publications, reports, and theses revealed that various fungal species were employed in mycofiltration, and species from the *Pleurotus* genus were predominant. Mycofiltration was performed in continuous flow, batch, or batch sequential mode, and a broad range of contaminants were mycofiltered from different water sources/matrices. Water sources included natural surface waters, real and synthetic wastewater, stormwater, and spiked tap/deionised water. Of the microbial contaminants, *E. coli* was the most frequently mycofiltered, whilst pharmaceuticals were the most studied of the organic contaminants. Regarding inorganic contaminants, heavy metals were mostly assessed for removal by mycofiltration. Generally, removal efficiencies (R%) were high, ranging between 60% and 100%.

### 5.1. Implications for Policy/Management

The implications of mycofiltration for environmental policy and management are multiple. As documented herein, this technology offers an affordable means for sustainable pollution control, helping with the removal of diverse biological and chemical pollutants from water. Given its eco-friendliness, affordability, and relative ease of use, governments could integrate mycofiltration into water quality regulations, especially in the developing world, where widespread challenges related to water management exist. To aid in valorising agricultural and forestry waste, this technology can be deployed using such waste as substrates, thus creating a closed-loop system that can add value to waste management and the circular economy. Policymakers can help integrate mycofiltration into organic waste recycling regulations, making it a fungus-based approach to biowaste valorisation. These potential next-generation applications of mycofiltration represent emerging research frontiers that require ample funding.

Indeed, promising innovations are still to emerge from the use and application of mycofiltration in real-world settings (i.e., in situ, industrial applications), as this intervention finds more relevance in both biotechnology and ecology. From the studies reviewed herein, despite promising capabilities in contaminant removal, the lack of real-world validation limits confidence in the technology’s adoption in regulatory frameworks. Therefore, there is a pressing need for funding mycoremediation research and development. Public research grants, public–private partnerships, and living-laboratory endeavours focused on the study, development, and promotion of mycoremediation techniques will ensure that this technology is exploited adequately through expansion beyond bench-scale research.

### 5.2. Implications for Research

The present systematic review observed some research gaps. The studies reviewed were all laboratory-based; however, there were no standard methods used in the preparation or design of the mycofilters. Standardisation ensures consistency and reproducibility across different laboratories/studies. Additionally, standardised methods will allow direct comparison of results across studies and reduce heterogeneity. For future research, standardisation based on substrate use and mycofilter design would be beneficial. Regarding the substrates, this systematic review observed that the substrates used were of varying consistency, from dust/powder form to coarse/bulky particles (e.g., woodchips). The specific size and surface area of these particles were not highlighted. Surface area is an important metric, as it influences the efficiency and capacity of contaminant removal and thus should be reported. A standardised substrate grading system would thus play a significant role in ensuring that substrates that are similar in nature (e.g., sawdust and rice husks) are inoculated with mycelia and packed following one standard method. The layering and packing of the substrate (and subsequently the mycofilter), particularly in the continuous flow set-up, affects how the water being filtered trickles down the mycofilter, which ultimately determines the contaminant removal efficiency. Concerning mycofilter design, in the current review, different-sized jars, columns, buckets, etc., were used for loading the mycofilters. Having specific standards for setting up each of these would be of great importance with regard to calculating accurate metrics for scaling up the mycofiltration system. Laboratory studies largely serve as proof of concept and provide baseline information. Researchers could reliably build upon findings from such standardised experiments to upscale mycofiltration for implementation in the real world, i.e., field/environmental settings and the industry.

The systematic review also observed that not all studies reported on mycofiltration duration and almost none of the studies investigated mycofilter capacity, i.e., mycofilter saturation. Mycofilter capacity is an important metric, as it gives an indication of when a mycofilter has reached its full capacity, pending renewal or removal. This can be achieved by conducting mycofiltration following a time series, i.e., measuring the concentrations of the mycofiltered samples at time intervals until outlet and inlet concentrations become equal. For the continuous flow set-up, mycofilter performance is typically assessed by breakthrough curves, which are a representation of the pollutant–effluent concentration at the outlet of the mycofilter versus time. Towards saturation, the contaminant concentration begins to increase in the effluent until no further removal takes place (the column is then saturated/exhausted), and the ratio of outlet concentration/inlet concentration (C/C0) is 1.

In this review, it was observed that little was done in exploiting the metabolically active mycelia’s ability to adapt and recover from abiotic stressors. Future studies could investigate the efficiency of spent/used mycofilters subjected to a period of recovery and rejuvenation. Having adapted to the previous stressor, a recovered mycofilter could be more efficient in contaminant removal upon repeated exposure to the same stressor. This process of mycofilter recovery and rejuvenation could also potentially lead to end-of-life valorisation, further integrating mycoremediation into circular economy frameworks. These mycofilters could be converted to biochar- or mycelium-based composite materials. However, before valorisation can be achieved, waste hazard and ecotoxicity assessments of such mycofilters must be conducted.

## Figures and Tables

**Table 1:** Criteria used to assess study validity.

Criterion	Checklist Question	Low Validity	Medium Validity	High Validity
Study design	Was there a sufficient number of replicates?	Fewer than two replicates	Two replicates	Three or more replicates
Risk of performance bias	Was there a clear link between hypothesis and measured outcomes?	No link between hypothesis and measured outcomes	Clear enough link between hypothesis and measured outcomes	Clear link between hypothesis and measured outcomes
Risk of reporting bias	Was the reported effect estimate based on selected measurements of the outcome?	Effect estimate was based on some of the measured outcomes (subgroup of <50% of all measured outcomes)	Effect estimate was based on some of the measured outcomes (subgroup of 50% of all measured outcomes)	Effect estimate was based on all of the measured outcomes
	Was there clarity of measured outcomes before vs. after intervention (i.e., no missing data)?	Measured outcomes before vs. after not adequately stated	Measured outcomes before vs. after clear enough	Measured outcomes before vs. after clearly and adequately stated
Risk of analysis bias	Was there sufficient description of statistical analysis and results?	No clear/appropriate description of statistical analysis or results	Clear enough description of statistical analysis and results	Clear and appropriate description of statistical analysis and results

**Table 2:** Data coding employed for data extraction.

Code	Variable	Description
Bibliographic information	Author	Only last name of the first author, and et. al., for the other colleagues.
	Publication year	Year the paper was published.
	Title	Full title of the paper.
	Source of publication	Nature of the publication, e.g., journal article, report, etc.
Study location	Country	The country where the study was conducted.
Study site	Field/laboratory	If the study was based in the field or laboratory.
Seasonality	Season	The season(s) in which the study was carried out, e.g., summer, winter, etc.
Water source	Sample nature	Nature of the water sample subjected to filtration, e.g., river water, stormwater, effluent, etc.
Fungal species	Species name	Fungal species employed in the filtration study, e.g., *P. ostreatus*.
Substrate employed	Substrate	Nature of the substrate used to create mycofilter, e.g., wheat straw.
Types of contaminants removed	Organic contaminants	Specific organic contaminants reported, e.g., pesticides.
	Inorganic contaminants	Specific inorganic contaminants reported, e.g., heavy metals.
	Microbial contaminants	Specific bacterial contaminants reported, e.g., *E. coli*.
	Contaminant limit of detection	Lowest concentration of the contaminant that could be reliably detected.
Mycofiltration process duration	Time period	The total period of time the mycofiltration process ran from start to finish (minutes, hours, days, etc.).
	Flow rate	Flow rate used for continuous flow experiments.
Mycofilter unit design and set-up	Design	Experimental design, stating if it was a single- or double-unit filter, etc.
	Mass	Weight of mycofilter in grams and/kilograms
	Set-up	Stating the nature of the set-up, whether it was a continuous flow or a batch system.
Reported levels of contaminants pre- and post-mycofiltration	Contaminant levels pre-mycofiltration	Concentrations/levels expressed in the units as reported in the study.
	Contaminant levels post-mycofiltration	Concentrations/levels expressed in the units as reported in the study.
Comparator	Control	Nature of the study/experiment control, i.e., no fungal filter.

## Data Availability

The original contributions presented in this study are included in the article/Supplementary Materials. Further inquiries can be directed to the corresponding author.

## Supplementary Materials

The following supporting information can be downloaded at https://www.mdpi.com/article/10.3390/jof12050376/s1. References [1,2,14,16,17,19,39] are cited in Supplementary File S2.
