# Supplementary material for: Mycofilters and the Effectiveness of Mycofiltration in the Removal of Contaminants in Water—A Systematic Review
Source: J Fungi (Basel). 2026 May 18;12(5):376. doi: 10.3390/jof12050376 (PMC13209015; doi:10.3390/jof12050376)
Supplement: Supplementary file 1 [file jof-12-00376-s001.zip › Supplementary file S1_Search strings_01 Apr 2026.pdf]

Search strings

This file presents a list of search strings used and formatted for the ScienceDirect, Web of Science and Scopus databases and Google internet search.

Keywords and hashtags used in searching organisation websites and social and research networking sources are also listed.

Science direct database:

String 1 = (“mycofilter” OR “fungal filter” OR “fungal filtration”) AND (“river pollution” OR “contaminated water” OR “polluted water” OR “stormwater” OR "wastewater”)

String 2 = (“mycofiltration” OR “fungal filter” OR “fungal filtration”) AND (“river pollution” OR “contaminated water” OR “polluted water” OR “stormwater” OR "wastewater”)

String 3 = (“mycofilter” OR “fungal filter” OR “fungal filtration”) AND (“borehole pollution” OR “dam pollution” OR “pond pollution” OR “rainwater” OR "effluent”)

String 4 = (“mycofiltration” OR “fungal filter” OR “fungal filtration”) AND (“borehole pollution” OR “dam pollution” OR “pond pollution” OR “rainwater” OR "effluent”)

String 5 = (“mycofilter” OR “fungal filter” OR “fungal filtration”) AND (“untreated water” OR “treated water”)

String 6 = (“mycofiltration” OR “fungal filter” OR “fungal filtration”) AND (“untreated water” OR “treated water”)

String 7 = (“fungal bioremediation” OR “mushroom filter” OR “mycelial filter” OR “mycelium colonised”) AND (“river pollution” OR “contaminated water” OR “polluted water” OR “stormwater” OR "wastewater”)

String 8 = (“fungal bioremediation” OR “mushroom filter” OR “mycelium filter” OR “mycelium colonised”) AND (“river pollution” OR “contaminated water” OR “polluted water” OR “stormwater” OR "wastewater”)

String 9 = (“fungal bioremediation” OR “mushroom filter” OR “mycelia filter” OR “mycelium colonised”) AND (“river pollution” OR “contaminated water” OR “polluted water” OR “stormwater” OR "wastewater”)

String 10 = (“fungal bioremediation” OR “mushroom filtration” OR “mycelial filter” OR “mycelium colonised”) AND (“borehole pollution” OR “dam pollution” OR “pond pollution” OR “rainwater” OR "effluent”)

String 11 = (“fungal bioremediation” OR “mushroom filtration” OR “mycelium filter” OR “mycelium colonised”) AND (“borehole pollution” OR “dam pollution” OR “pond pollution” OR “rainwater” OR "effluent”)

String 12 = (“fungal bioremediation” OR “mushroom filtration” OR “mycelia filter” OR “mycelium colonised”) AND (“borehole pollution” OR “dam pollution” OR “pond pollution” OR “rainwater” OR "effluent”)

String 13 = (“fungal bioremediation” OR “mushroom filtration” OR “mycelial filter” OR “mycelium colonised”) AND (“untreated water” OR “treated water”)

String 14 = (“fungal bioremediation” OR “mushroom filtration” OR “mycelium filter” OR “mycelium colonised”) AND (“untreated water” OR “treated water”)

String 15 = (“fungal bioremediation” OR “mushroom filtration” OR “mycelia filter” OR “mycelium colonised”) AND (“untreated water” OR “treated water”)

Web of Science database:

TS= (mycofilt* OR fungal filter OR fungal filtration OR fungal bioremediation OR mushroom filtration OR myceli* filter OR mycelium colonised) AND TS= (borehole pollution OR river pollution OR dam pollution OR pond pollution OR contaminated water OR polluted water OR stormwater OR rainwater OR wastewater OR effluent OR untreated water OR treated water).

Scopus database, Google Scholar internet search:

(“mycofilt*” OR “fungal filter” OR “fungal filtration” OR “fungal bioremediation” OR “mushroom filtration” OR “myceli* filter” OR “mycelium colonised”) AND (“borehole pollution” OR “river pollution” OR “dam pollution” OR “pond pollution” OR “contaminated water” OR “polluted water” OR “stormwater” OR “rainwater” OR “wastewater” OR “effluent” OR “untreated water” OR “treated water”)

Keywords for Open Access Theses and Dissertation, and organisation websites:

Mycofiltration, mycofilter, mushroom filter, fungal filtration, fungal bioremediation

Keywords and #tags for social and research networking sources:

X (Twitter): #mycofiltration, #mycofilter, #mushroomfilter, #fungalfiltration, #fungalbioremediation

Facebook: mycofiltration, mycofilter, fungal bioremediation

ResearchGate: mycofiltration, mycofilter, fungal bioremediation.
