# Supplementary material for: Mycofilters and the Effectiveness of Mycofiltration in the Removal of Contaminants in Water—A Systematic Review
Source: J Fungi (Basel). 2026 May 18;12(5):376. doi: 10.3390/jof12050376 (PMC13209015; doi:10.3390/jof12050376)
Supplement: Supplementary file 1 [file jof-12-00376-s001.zip › Supplementary file S2_Benchmark articles_01 Apr 26.pdf]

README

Benchmark articles

This file presents a list of benchmark articles (key scientific papers) of known relevance to mycofiltration. These should be returned, following a search using the search string, to determine the comprehensiveness of the search.

List of benchmark articles:

1. Akpaj E and Olorunfemi D. Mycofiltration effectiveness in bioremediation of contaminated drinking water sources. Ife Journal of Science. 2014; 16(3).

2. Olorunfemi D, Efechuku U, and Esuana J. Toxicological Evaluation of Drinking Water Sources in Some Rural Communities in Southern Nigeria after Mycofiltration Treatment. Pol J Environ Stud. 2015; 24.

3. Olorunfemi Daniel, Uzakah Richard, Ofomata Romeo & Charles, O. 2020. Evaluation of toxicity and bioremediation of contaminated drinking water sources in delta state, nigeria. J Adv Biol Biotechnol*,* 23**,** 8-16.

4. Osarenotor, O., H. Essandoh, and I. Aighewi, Removal of pollutants by mycelium colonized sawdust. Water Pract Technol, 2021.

5. Pini A and Geddes P. Fungi Are Capable of Mycoremediation of River Water Contaminated by E. coli. Water Air Soil Pollut. 2020; 231.

6. Taylor A, Flatt A, Beutel M, Wolff M, Brownson K, and Stamets P. Removal of Escherichia coli from synthetic stormwater using mycofiltration. Ecol Eng. 2015; 78 79-86.

7. Vu NN, Ngoc PVK and Hoa PT. Antibacterial activity of three wild wood-decaying fungi in southern Vietnam toward *Vibrio parahaemolyticus* bacterium in aquaculture wastewater. Vietnam Journal of Biotechnology. 2016; 14(4).
